# Temporal Features of Psychological and Physical Self-Representation: An ERP Study

**DOI:** 10.3389/fpsyg.2019.00785

**Published:** 2019-04-10

**Authors:** Lei Liu, Wenjie Li, Jin Li, Lingna Lou, Jie Chen

**Affiliations:** ^1^ School of Educational Science, Hunan Normal University, Changsha, China; ^2^ Cognition and Human Behavior Key Laboratory, Hunan Normal University, Changsha, China; ^3^ School of Psychological and Cognitive Science, Peking University, Beijing, China; ^4^ Faculty of Philosophy, Martin Luther University Halle-Wittenberg, Halle, Germany

**Keywords:** psychological-self, physical-self, own name, own voice, P3, N400

## Abstract

Psychological and physical-self are two important aspects of self-concept. Although a growing number of behavioral and neuroimaging studies have investigated the cognitive mechanism and neural substrate underlying psychological and physical-self-representation, most of the existing research on psychological and physical-self-representation had been done in isolation. The present study aims to examine the electrophysiological responses to both psychological (one’s own name) and physical (one’s own voice) self-related stimuli in a uniform paradigm. Event-related potentials (ERPs) were recorded for subjects’ own and others’ names uttered by subjects’ own or others’ voice (own voice-own name, own voice-other’s name, other’s voice-own name, other’s voice-other’s name) while subjects performed an auditory passive oddball task. The results showed that one’s own name elicited smaller P2 and larger P3 amplitudes than those of other’s names, irrespective of the voice identity. However, no differences were observed between self and other’s voice during the P2 and P3 stages. Moreover, an obvious interaction effect was observed between voice content and voice identity at the N400 stage that the subject’s own voice elicited a larger parietal N400 amplitude than other’s voice in other name condition but not in own name condition. Taken together, these findings suggested that psychological (one’s own name) and physical (one’s own voice) self-representation induced distinct electrophysiological response patterns in auditory-cognitive processing.

## Introduction

Self-recognition is an essential biological and social function for human species, which represents a capacity to identify the distinction between self and others (Candini et al., 2014; van Veluw and Chance, 2014; Conde et al., 2015). Multiple behavioral and neuroimaging studies have revealed the cognitive and neural mechanisms of self-reference effect (Kalenzaga et al., 2015; Humphreys and Sui, 2016; Tamura et al., 2016), self-relevant effect (Chen et al., 2011, 2015a,b), and self-positive bias (Watson et al., 2007; Fields and Kuperberg, 2015; Kiang et al., 2017). However, these studies usually characterized the self at a single and unidimensional structure (Platek et al., 2004). From the time of William James, the multidimensionality of self-concept has been emphasized and highlighted (Marsh, 1990), some psychologists suggest that the self-concept cannot be understood sufficiently and accurately if its multidimensionality is ignored (Marsh, 1990; Walla et al., 2008; Klein, 2010; Uddin, 2011; Philippi et al., 2017). For example, Gillihan and Farah (2005) reviewed a series of self-related studies and divided the self-concept into two aspects: physical and psychological self (Gillihan and Farah, 2005). The physical-self contains the sensory and image-based representations of our face, voice, proprioceptive and motor-based representations of our body (Gallagher, 2005; Gillihan and Farah, 2005; Keyes et al., 2010; Uddin, 2011; Sugiura, 2013), which is the biological basis of the self. The psychological-self involves the processing of self-related knowledge (e.g., autobiographical memory, semantic memory knowledge about oneself) and the first-person perspective, such as personality trait adjectives, own name, own born place (Gray et al., 2004; Gillihan and Farah, 2005; Su et al., 2010; Uddin, 2011; Tateuchi et al., 2015). Moreover, Kircher et al. (2000) suggested that the physical and psychological self-processing might involve different cognitive and neural mechanism (Kircher et al., 2000). Hu et al. (2016) examined the commonalities and distinctions between physical and psychological self-representation using an ALE meta-analysis. They found that physical self-representation was particularly linked to lateral brain regions with a right hemispheric dominance, while psychological self-representation significantly and predominantly activated the cortical midline structures. Moreover, the anterior cingulate cortex (ACC) and left inferior frontal gyrus (IFG) were activated both in physical and psychological self-processing (Hu et al., 2016).

However, most of these existing studies on physical- and psychological-self had been done in isolation, and there was relatively limited research directly examing the physical and psychological self-processing in the same study. Kircher et al. (2000) firstly studied the neural correlates of physical (self-face) and psychological (self-related trait) self-processing using the functional magnetic resonance imaging (fMRI). Their results showed that physical self-processing is related to the right limbic areas, right middle temporal lobe, left inferior parietal, and the left prefrontal regions, while the psychological-self activated the precuneus, left parietal lobe, left insula/IFG, and the left ACC (Kircher et al., 2000). Lou et al. (2004) further found that the right lateral parietal regions not only played an important role in physical-self (e.g., imagination of agency, body representation) but also activated in the psychological-self task (Lou et al., 2004). Although these studies have advanced our understanding of the similarities and differences in terms of the neural mechanism underlying physical and psychological self-related processing, little is known about the dynamical temporal features of physical and psychological self-related processing.

With the advantages of high temporal resolution and direct measure of neural activity, event-related potential (ERP) is an ideal methodology for exploring the dynamical temporal features of cognitive processing (Graux et al., 2013, 2015; Pinheiro et al., 2016, 2017). A growing number of ERP studies have provided evidences of the self-relevant effect. For example, the P3 component could be the most noticeable marker of the self-relevant effect, larger P3 amplitude was almost always elicited by self-relevant stimulus (e.g., the subject’s own name) than non-self-relevant stimulus (Berlad and Pratt, 1995; Gray et al., 2004; Chen et al., 2013; Humphreys and Sui, 2016). Recent studies also have suggested that the N400 component is sensitive to the meaning processing and memory retrieval of self-concept (Metzler et al., 2014; Fields and Kuperberg, 2015; Coronel and Federmeier, 2016). In addition, the P2 component may also index privileged automatic process of self-related stimulus. Thus, the current study uses ERP measures to investigate the dynamical temporal course of physical- and psychological self-related processing.

Four types of vocal stimuli (subject’s own name and other’s name uttered by subject’s own and other’s voices) were recorded as the physical/non-physical and psychological/non-psychological self-related stimuli. We examine the psychological self-relevant effect by comparing the difference between subject’s own and other’s name (Gray et al., 2004; Gillihan and Farah, 2005; Su et al., 2010), while we examine the physical self-relevant effect by comparing the difference between subject’s own and other’s voice (Su et al., 2010; Xu et al., 2013; Conde et al., 2015). Moreover, in order to set the experimental design similar to natural situation, our study adopted an oddball paradigm, and the setting of experimental stimuli can well match the stimulus and task properties during physical- and psychological self-related processing. Four types of vocal stimuli were presented as rare and task-irrelevant stimuli. Participants were instructed to distinguish and recognize an 800 Hz pure tone from 1,000 Hz pure tone standard stimuli. Besides, we also conducted a control task with two unfamiliar other’s names uttered by two unfamiliar other’s voices as small probability and task-irrelevant stimuli, the control task aims to test the controlling effect of unrelated acoustic properties (such as the F0, see Graux et al., 2013; Conde et al., 2015).

Thus, as previous neuroimaging studies demonstrating differences in terms of the neural mechanisms underlying physical and psychological self-processing (Kircher et al., 2000; Lou et al., 2004; Hu et al., 2016), we hypothesized that the physical and psychological self-processing might also elicit different electrophysiological responses in P2, P3, or N400 components, which were sensitive to different types of self-relevant processing (Chen et al., 2011, 2013, 2015a; Metzler et al., 2014; Fields and Kuperberg, 2015). We also hypothesized that there is no statistically significant effect in each condition of control task.

## Materials and Methods

### Participants and Design

Thirty-one undergraduates (eight males), age between 18 and 25 years (*M* = 21.3 years), participated in the experiment. All participants were native Mandarin speakers without any local accent, right-handed, with normal hearing, normal or corrected-to-normal vision, and no history of neurological or psychiatric disorders. Each participant was given written informed consent prior to the experiment and received a certain amount of money for their reward after the experiment. The experiment was conducted in accordance with the Declaration of Helsinki and approved by the Ethics Committee of Liaoning Normal University. The experiment includes two tasks, and all subjects participated in the experimental task, while only 25 subjects participated in the control task due to the long time interval. Besides, one subject’s nose reference was invalid in whole experimental procedure, and the valid trials of two subjects were too less (invalid trials >25%) to accepted in control task. Thus, only 30 valid subjects in experimental task and 22 valid subjects in control task are included in the ERP analysis.

### Stimuli and Materials

A pool of voices was recorded by Philips SHM1000 microphone^1^ with Adobe Audition CS6 (Adobe Systems Inc., San Jose, CA, USA) at a sampling rate of 44,100 Hz and16-bit resolution 1–2 weeks before the ERP experiment. We created a voice template by a trained female speaker before the recording. Each participant was instructed to pronounce names (subject’s own name and names of other subjects in the present study) according to the voice template with neutral prosody, and each name was repeated 10 times. All names are three Chinese characters. The voice materials were further edited to 600 ms in duration and with sound intensity in 70 dB by Audition CS6 and Praat^2^. Finally, four types of vocal stimuli (subject’s own name and other’s name uttered by subject’s own and other’s voices) were selected for the experiment. Voice stimuli were gender-matched and have similar mean fundamental frequency (F0) for each participant.

### Procedure

An auditory passive oddball paradigm was used in the present study, in which the 1,000-Hz pure-tone was presented 600 times (60%), the 800-Hz pure-tone was presented 80 times (8%), and each set of voice stimuli 80 times (8%). Each trial began with a fixation cross presented for 200 ms, followed by an intertrial interval (ITI) of 300–500 ms. Then, the experimental stimuli (voice stimuli, 800-Hz and 1,000-Hz pure-tones) were presented for 600 ms. The task of the participants was to detect the 800-Hz pure-tone and to press the “F” key on the keyboard with their right index finger if the 800-Hz pure-tone was presented. No responses were required for other stimuli. Each experimental stimulus was followed by a blank with 800 ms duration. There were totally five blocks, and the sequence of stimuli was randomized across conditions in each block.

**Figure 1:**
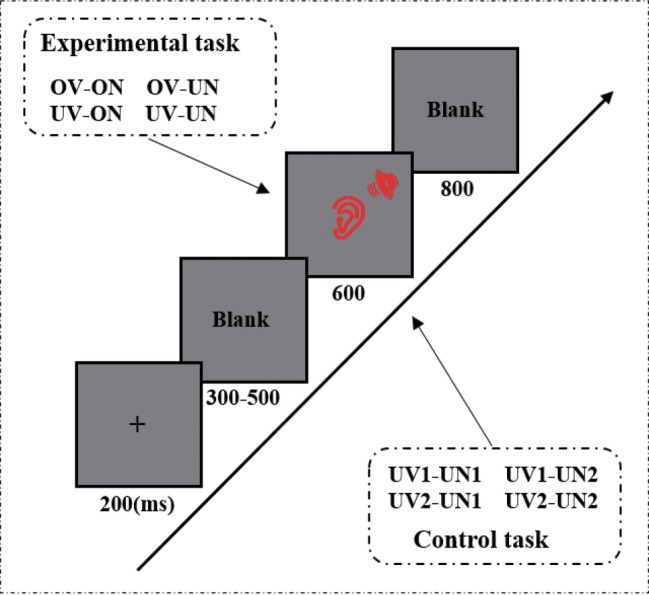
An illustration of the oddball paradigm sequence in a single trial (including experimental task and control task). The only difference between experimental task and control task is that the rare and task-irrelevant stimuli. The experimental task used the ov-on, ov-un, uv-on, and uv-un as task-irrelevant stimuli, but the control task adopted the uv1-un1, uv1-un2, uv2-un1, and uv2-un2 as task-irrelevant stimuli.

The whole experiment includes the experimental and control task. The experimental procedures for the two tasks were identical except the set of voice stimuli. During the experiment task, the voice stimuli were one’s own name uttered by his/her own voice (ov-on), one’s own name uttered by unfamiliar other’s voice (uv-on), unfamiliar other’s name uttered by own voice (ov-un), and unfamiliar other’s name uttered by unfamiliar other’s voice (uv-un). During the control task, the voice stimuli were two unfamiliar other’s names uttered by two unfamiliar people (uv1-un1, uv1-un2, uv2-un1, and uv2-un2) (see Figure 1). Participants were required to do eight trial exercises to familiarize the 800-Hz and 1,000-Hz pure-tones before the formal experiment. Stimulus presentation was accomplished with E-prime 2.0 software (Psychology Software Tools, Pittsburgh, PA, USA).

## Electrophysiological Recording and Data Analysis

### EEG Recording

Continuous EEG signals were recorded using an “EEGo Sports” EEG system (ANT Neuro, Enschede, the Netherlands) with 65 Ag/AgCl electrodes arranged in an international 10/10 system layout. Additional electrodes were applied on the nose for offline analysis, above and below the left eye to record the electro-oculogram (EOG). The signals were recorded with a sampling rate of 500 Hz. The online reference is CPz and the impedance of each electrode was maintained below 5 kΩ. The EEG data were re-referenced offline to the nose and filtered at 0.1–30 Hz by Butterworth filter.

### Data Analysis

The EEG data were pre-processed by the EEGLAB toolbox (Delorme and Makeig, 2004). We visually inspected the EEG data and removed trials containing high amplitude noise, such as large body movements-related high-frequency noise and other easily identifiable confounds such as sudden electrode drifts and jumps. Then, the eye-blinks, saccades, and any other consistent artifacts were removed using independent component analysis (Delorme and Makeig, 2004). Bad channels were interpolated based on the data of neighboring electrodes. And the continuous data were epoched into single trials beginning 200 ms before sound stimuli presentation and ending 800 ms after stimuli presentation. The data were baseline-corrected according to the 200 ms before the onset of sound stimuli. ERP trials with residual artifacts (mean voltage exceeding ±80 μV) were excluded from averaging, and if the number of artifact trials is more than 25% of the total trials, the subject was deleted. Artifact-free ERP trials were averaged separately for each experimental condition.

After a cautious inspection of the topographic maps and grand-averaged ERP waveforms (see Luck and Gaspelin, 2017; Jack et al., 2019), a central N1 (averaged of C3/z/4) in the time-window of 110–210 ms, central P2 (averaged of C3/z/4) in the time-window of 210–310 ms, parietal P3 (averaged of P3/z/4) in the time-window of 310–410 ms, and parietal N400 (averaged of P3/z/4) in the time-window of 420–480 ms were identified. These scalp areas and time-windows are also consistent with the previous literature (see Duncan et al., 2009; Kutas and Federmeier, 2011; Stekelenburg and Vroomen, 2012). Consistent with the experimental task, a scalp central area (averaged of C3/z/4) for the time-window 110–210 ms and 210–310 ms was selected in the control task. As no prominent late components were elicited in the control task, a parietal area (averaged of P3/z/4) for the time-window 310–410 ms and a frontal area (averaged of F3/z/F4) for the time-windows 410–510 ms and 510–610 ms were selected according to the collapsed localizer methods (Luck and Gaspelin, 2017). A two-way repeated measures analysis of variance (ANOVA) was performed on all measured amplitudes for each component. ANOVA factors were the voice identity (two levels: own voice vs. unfamiliar other’s voice), voice content (two levels: own name vs. unfamiliar other’s name). The ERP results were calculated by the ERPLAB toolbox (Lopezcalderon and Luck, 2014), and the statistical analysis was conducted by the IBM SPSS 20.0 (IBM Corp., Armonk, NY, USA). The degree of freedom of F-ratios was corrected according to the Greenhouse-Geisser method. The Bonferroni correction was used in multiple comparison correction, and the partial eta-squared ($\eta_{p}^{2}$) was reported as a measure of effect size.

## Results

### The Results of Experimental Task

**Figure 2:**
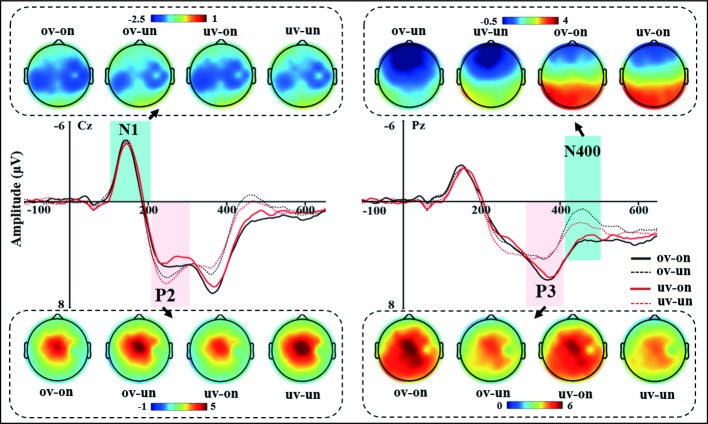
The grand-averaged ERP waveforms at Cz and Pz and the topographic maps of N1, P2, P3, and N400 for four experimental conditions. The light blue bar of left panel highlighted the time-window of the N1 (110–210 ms), no any significant differences were found. The light pink bar of left panel highlighted the time-window of the P2 (210–310 ms). Difference in P2 mean amplitudes between psychological-self and non-psychological-self are shown in the left panel. The light pink bar of right panel highlighted the time-window of the P3 (310–410 ms), difference in P3 mean amplitudes between psychological- and non-psychological-self are also shown in right panel. The light blue bar of right panel highlighted the time-window of the N400 (420–480 ms). Difference in N400 means amplitudes between physical- and non-physical-self are also shown in right panel.

The grand-averaged ERP waveforms of four conditions (ov-on, ov-un, uv-on, and uv-un) were illustrated in Figure 2, with scalp voltage topographical maps for N1, P2, P3, and N400 components.

**N1:** The left panel of Figure 2 shows the grand-averaged ERP waveforms and scalp voltage topographic maps of central N1 component for each condition at Cz electrode. There was neither a significant main effect for the voice identity, *F*_(1,29)_ = 0.006, *p* = 0.941, nor for the voice content, *F*_(1,29)_ = 0.598, *p* = 0.446. There was also no interaction effect between voice identity and voice content (*F*_(1,29)_ = 0.061, *p* = 0.807). These results revealed a similarity of early auditory processing of the four types voice stimuli.

**P2:** The two-way repeated ANOVA measures for mean amplitude of central P2 component demonstrated significant main effect of voice content (*F*_(1, 29)_ = 6.496, *p* = 0.016, $\eta_{p}^{2}$ = 0.183), own name (3.036 μV) elicited smaller P2 amplitude than other’s name (3.641 μV). However, there was neither a significant main effect for voice identity (*F*_(1, 29)_ = 0.030, *p* = 0.864) nor significant interaction effect for voice identity and voice content (*F*_(1, 29)_ = 1.775, *p* = 0.193) (see Figure 2).

**P3:** The grand-averaged ERP waveforms of P3 at Cz and Pz electrodes and topographic maps of parietal P3 for each condition were showed in Figure 2. The two-way repeated ANOVA results showed a significant main effect for voice content (*F*_(1, 29)_ = 10.399, *p* = 0.003, $\eta_{p}^{2}$ = 0.264). Compared to the unfamiliar other’s name (3.761 μV), subject’s own name (4.941 μV) elicited larger P3 amplitude. It means that psychological-self elicited a larger P3 component than non-psychological-self. However, the main effect of voice identity (*F*_(1, 29)_ = 0.032, *p* = 0.860), the interaction effect between voice identity and voice content (*F*_(1,29)_ = 0.121, *p* = 0.731), did not reach significance.

**N400:** The two-way repeated ANOVA results of parietal N400 component demonstrated a significant main effect of voice content (*F*_(1, 29)_ = 18.737, *p* < 0.001, $\eta_{p}^{2}$ = 0.393); unfamiliar other’s name (1.450 μV) elicited more negative N400 amplitude than subject’s own name (3.165 μV). More importantly, the interaction between voice identity and voice content was also significant, *F*_(1, 29)_ = 4.691, *p* = 0.039, $\eta_{p}^{2}$ = 0.139. The further simple main effect analysis revealed that there is no difference between subject’s own voice and other’s voice under the own name condition (*F*_(1, 29)_ = 0.191, *p* = 0.665), while own voice (1.076 μV) elicited more negative N400 amplitude than other’s voice (1.823 μV) under the unfamiliar other’s name condition, *F*_(1, 29)_ = 5.048, *p* = 0.032 (see Figure 2). Besides, the main effect of voice identity failed to reach significance, *F*_(1, 29)_ = 2.061, *p* = 0.162.

Taken together, these results indicated that psychological-self elicited a small P2, large P3, and N400 component than non-psychological-self. More importantly, there is an interaction effect between voice identity and voice content, specifically, physical-self (own voice) elicited a more negative N400 component than non-physical-self (other’s voice) under the non-psychological-self condition.

### The Results of Control Task

**Figure 3:**
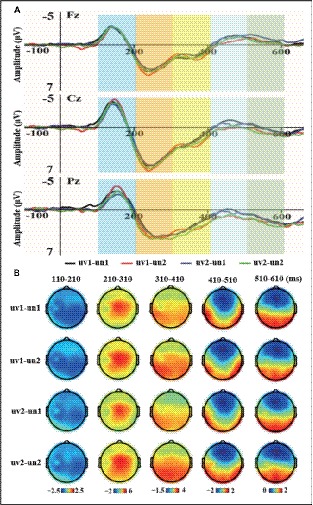
The grand-averaged ERP waveforms at Fz, Cz, and Pz and the scalp voltage topographic maps of five time-windows for four conditions. **(A)** The grand-averaged ERP waveforms with different color bars for five time-windows were shown in the upper panel. **(B)** The scalp voltage topographic maps of five time-windows for four conditions were shown in the bottom panel.

The two-way repeated ANOVA measures for mean amplitudes of the five time-windows were performed. The grand-averaged ERP waveforms of four conditions (uv1-un1, uv1-un2, uv2-un1, and uv2-un2) were illustrated in Figure 3A, with scalp topographical voltage maps for the five time-windows (see Figure 3B). The results showed that there was neither a significant main effect for the voice identity (*Fs* < 0.582, *ps* > 0.454) nor for the voice content (*Fs* < 4.191, *ps* > 0.053) during the five time-windows. Besides, no significant interaction effect was observed between voice identity and voice content during the five time-windows (*Fs* < 1.267, *ps* > 0.273) (Figure 3).

## Discussion

The present study examined the dynamical temporal features of the physical-self and psychological-self representation using ERP measures. Our results showed that subjects’ own name elicited smaller P2 and larger P3 amplitudes compared to other’s name, irrespective of the voice identity. However, no P2 and P3 differences were observed between subjects’ own voice and other’s voice until the late N400 stage, in which a significant voice identity by voice content interaction effect was observed. Subject’s own voice elicited more negative N400 amplitude than other’s voice under the other’s name condition, and no such effect was observed under own name condition. Thus, in addition to previous fMRI studies showing the different neural representation underlying the physical-self and psychological-self (Kircher et al., 2000; Hu et al., 2016), the present study further demonstrated different temporal features of physical-self and psychological-self representation using ERP measures.

The N1 component reflects the early auditory processing of the physical properties of the voice stimulus (Naatanen and Picton, 1987). In our current study, neither main effects nor interaction effects were observed at N1 stage perhaps due to the mean fundamental frequency (F0) was matched, and the voice intensity and duration were identical for the four types of voices for each participant.

Meanwhile, the present study found that subject’s own name elicited a smaller P2 component than unfamiliar other’s name, irrespective of the voice identity. It has been suggested that the P2 component reflects a selective attention (Chen et al., 2015b, 2017), a small P2 component may reflects an automatic attention. Minati et al. (2010) also confirmed that a melody-like sound sequence elicited a reduced P2 component than a random-generated sound sequence (Minati et al., 2010). Similar effects were also found in the Chinese phonograms processing. Hsu et al. (2009) found that characters with high combinability and high consistency elicited smaller P2 amplitude than low combinability and low consistency characters (Hsu et al., 2009). The high combinability and consisting obtained a more automatic process, which demonstrated a less positive P2 effect. Analogously, unlike unfamiliar other’s name that processed in phoneme unit, subject’s own name may be processed in syllable unit due to the binding effect of self (Sui and Humphreys, 2015). The decreased amplitude observed for own name suggest an easier detection and processing of the own name relative to the other’s name in auditory presented condition and indicates that psychological-self and non-psychological-self was discriminated at an early auditory attentional processing stage.

More importantly, our results demonstrated that subject’s own name elicited a larger P3 component than unfamiliar other’s name, irrespective of the voice type. In other words, the psychological-self elicited a larger P3 component than non-psychological-self. As previous studies claimed that the P3 component is a significant index of psychological self-representation, significant P3 component was elcitied even when participants was passive hearing their own name under either sleep or minimally conscious state (Perrin et al., 1999, 2006). It has been suggested that the P3 is related to attentional resource allocation (Polich, 2007), the larger P3 amplitude of psychological-self suggest that the psychological self-recruits a larger amount of attention resource than non-psychological-self. Moreover, this finding is consistent with previous studies that used other psychological-self-related stimuli, such as autobiographical information (Berlad and Pratt, 1995; Gray et al., 2004; Chen et al., 2011) and possessive pronouns (Zhou et al., 2010). Thus, the larger P3 effect of psychological-self could be illustrated by the fact that psychological-self evoked enhanced saliency and motivational expression.

Moreover, there was an interaction effect observed between voice identity and voice content on the N400 component. Subject’s own voice elicited more negative N400 amplitude than other’s voice when the voice content was other’s name, whereas no difference was observed between self and other’s voices when the voice content was subject’s own name. In other word, an obvious self-voice effect (physical self-relevant effect) was occurred when the voice content was other’s name. The N400 component was first proposed by Kutas and Hillyard, which typically occurs between 200 and 500 ms and maximal over the scalp of central-parietal sites (Kutas and Hillyard, 1980; Kutas and Federmeier, 2011). Some studies suggest that the N400 reflects the semantic violation, which usually elicits more negative N400 amplitude (Baetens et al., 2011; Kiang et al., 2017). One more general opinion proposes that the N400 indexes the access to long-term memory (Kutas and Federmeier, 2011). When participants starting to detect and identify their own voice, the heard record voices are not totally same as their own voices heard naturally due to different sound conducting ways. Specifically, we hear recorded sounds *via* air conduction only, whereas hearing own natural voice *via* both air and bone conduction (Graux et al., 2013). Thus, subjects’ own voices detected during the experiment was not totally and absolutely consistent with those stored in long-term memory, which might contribute to the larger N400 for subjects’ own than others’ voices when voice contents were others’ names. However, no N400 differences were observed between subjects’ own and others’ voices when voice contents were subjects’ own names. It was more likely because that the preference of processing subjects’ own names (psychological-self) inhibited the processing of subjects’ own voices (physical-self).

Furthermore, using unfamiliar others’ names uttered by different unfamiliar others’ voices as voice stimuli, the control task showed no significant main effects or interaction effects during the 110–210, 210–310, 310–410, 410–510, and 510–610 ms time intervals. Moreover, all vocal stimuli have similar mean fundamental frequency (F0) for each participant. Thus, these results might reflect that the psychological self-related and physiological self-related effects observed in the experimental task was due to the self-relevance rather than the acoustic properties of these voice stimuli.

In conclusion, the results of this study suggest that the psychological self-effect appeared in both the early P2, P3, and late N400 stages, while the physiological self-effect did not appear until the late N400 stage. Consistent with previous neuroimaging studies, the present study demonstrated a different temporal pattern between physical and psychological self-representation.

## Ethics Statement

The experiment was conducted in accordance with the Declaration of Helsinki and was approved by the Ethics Committee of Liaoning Normal University. Each participant was given written informed consent prior to the experiment.

## Author Contributions

LL, JC, and JL designed the study. LL, JC, WL and JL wrote the the manuscript. LL, JC, WL and LNL carried out all data analyses. LL, LNL conducted data collection. All authors contributed to and approved the final version of the manuscript.

### Conflict of Interest Statement

The authors declare that the research was conducted in the absence of any commercial or financial relationships that could be construed as a potential conflict of interest.

## Footnotes

^1^https://www.philips.com.cn

^2^http://www.fon.hum.uva.nl/praat/
